# Malignant acanthosis nigricans with oral manifestations in a young female: a case report and literature review

**DOI:** 10.3389/fonc.2024.1459148

**Published:** 2024-09-24

**Authors:** Zijian Liu, Wuling Cao, Yang Liu

**Affiliations:** ^1^ Department of Periodontics, Stomatological Hospital of Xiamen Medical College & Xiamen Key Laboratory of Stomatological Disease Diagnosis and Treatment, Xiamen, China; ^2^ Department of Oral Medicine, Peking University School and Hospital of Stomatology & National Center for Stomatology & National Clinical Research Center for Oral Diseases & National Engineering Research Center of Oral Biomaterials and Digital Medical Devices, Beijing, China

**Keywords:** malignant acanthosis nigricans, malignancy, papillomatosis, velvety hyperplasia, gastric cancer, case report

## Abstract

**Introduction:**

Almost all cases of malignant acanthosis nigricans with oral manifestations occurred in older age groups. Here, we report a case of malignant acanthosis nigricans in a young female presented with chief complaints of oral mucosal hyperplasia, who had previously been diagnosed with allergies.

**Case presentation:**

A 30-year-old female developed oral hyperplasia and rash following seafood consumption, with subsequent resolution of the rash but persistent oral lesions and the appearance of pigmentation on her hands, neck, and axillae. Clinical examination revealed papillomatosis, hyperemia, and velvety hyperplasia in the oral cavity. Biopsy results confirmed papillary hyperplasia. Despite the patient’s belief in good health, she was advised to undergo further systemic examinations. Elevated serum tumor markers and histologic analysis of an endoscopic biopsy confirmed gastric cancer with duodenal infiltration, leading to the diagnosis of malignant acanthosis nigricans. Unfortunately, the patient passed away due to heart failure during chemotherapy treatment.

**Conclusions:**

The majority of patients with malignant acanthosis nigricans present with oral lesions before the underlying malignancy is detected, emphasizing the importance of timely comprehensive systemic examination. Furthermore, our case suggests that age may not be a restrictive factor for the development of malignant acanthosis nigricans, and the presence of a rash can potentially obscure the cutaneous manifestations associated with this condition.

## 1 Introduction

Acanthosis nigricans is a rare mucocutaneous disorder characterized by skin hyperpigmentation and thickening in specific regions, such as the neck, axillae, and groin (1). Additionally, some patients exhibit filiform growths on the oral mucosa (2), which bear resemblance to lesions induced by human papillomavirus.

There were several different classifications for acanthosis nigricans, with malignant acanthosis nigricans included in each of them (3–5). Malignant acanthosis nigricans is considered a paraneoplastic syndrome, associated with cancers of the stomach, ovary, bladder, breast, liver, and kidney (6). Mucous membrane involvement is indicative of the malignant form.

Here, we report a case of a patient presenting with filiform papillomas on oral mucosa who was ultimately diagnosed with malignant acanthosis nigricans. While malignant acanthosis nigricans has mainly been reported in older populations (7), the patient in this case was only 30 years old. She had confidence in her physical health and denied all relevant systemic symptoms at her initial visit.

## 2 Case report

A 30-year-old woman presented to the Oral Medicine Department of Peking University School and Hospital of Stomatology with a complaint of 4-month hyperplasia in the mouth. She recalled that the oral manifestation and skin eruptions occurred after consuming seafood and had previously been diagnosed with atopic dermatitis at other institutions. The rash resolved after treatment, whereas pigmentation appeared on the hands, neck, and axillae. However, there was no improvement in the oral lesions. The patient underwent laboratory tests (blood routine, blood biochemistry, erythrocyte sedimentation rate, ANA spectrum, and T-cell subtype), and the results were all within normal ranges. The patient denied weight loss, a history of cancer, drug use, diabetes, unconventional sexual contact, venereal disease, and other endocrine disorders.

**Figure 1:**
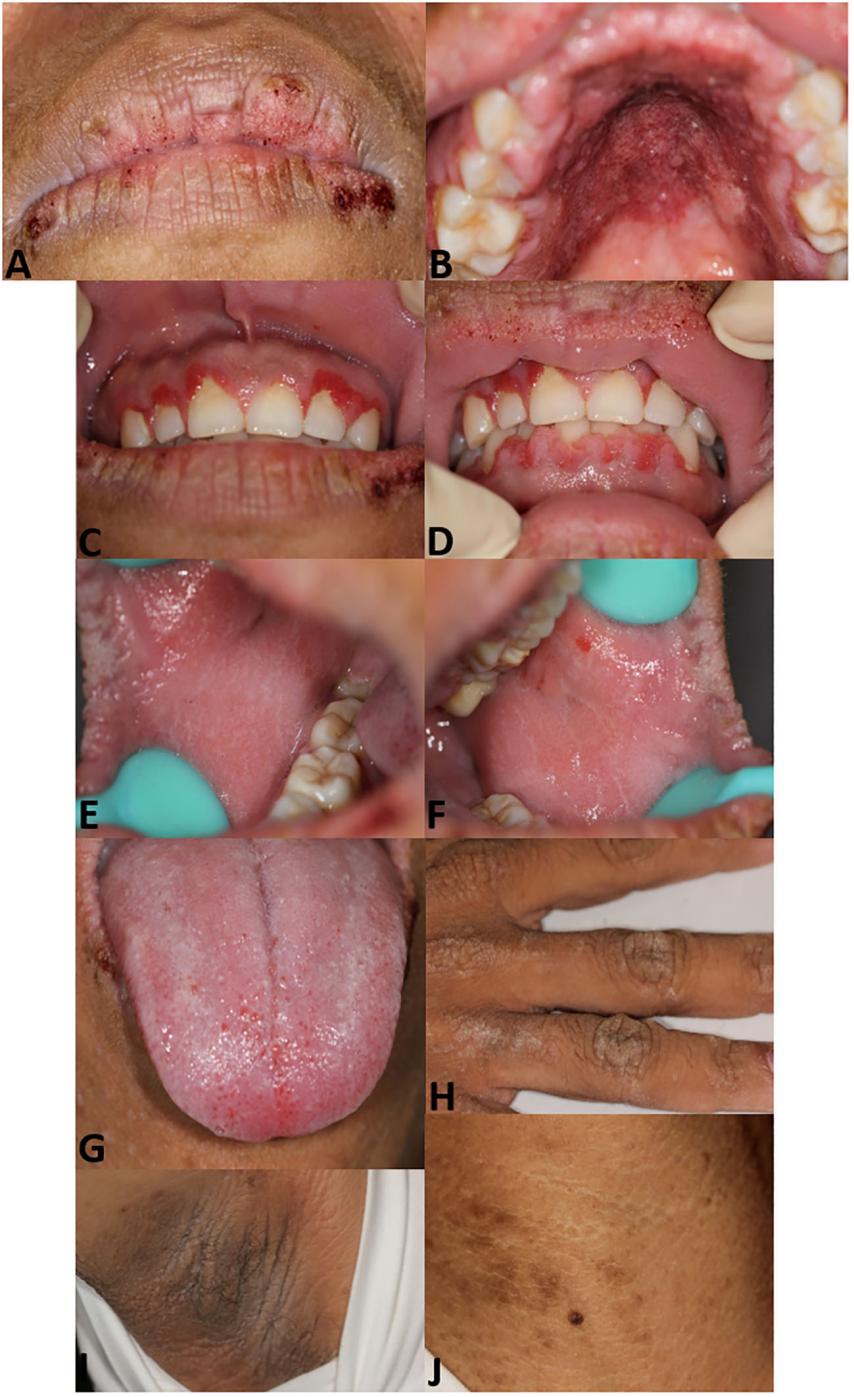
Patient images. Papillomatosis with scab on lips **(A)**. Papillomatosis on hard palate mucosa with hyperemia **(B)**. Papillomatosis on gingiva **(C, D)**. Velvety hyperplasia on right **(E)** and left buccal mucosa **(F)**. Velvety verrucous hyperplasia on the dorsum of the tongue and swelling of lingual papilla **(G)**. Hyperpigmentation on the hand **(H)**, axillae **(I)**, and neck **(J)**.

Oral examination results identified papillomatosis on the lips, hard palate mucosa, and gingiva with apparent hyperemia. Velvety hyperplasia was observed on the buccal mucosa and the dorsum of the tongue. Dermatological examination showed hyperpigmentation on the neck, axillae, and hands (**Figure 1**). Biopsies were performed on the lower lip and axillae, revealing papillary hyperplasia (**Supplementary Figure S1**).

**Figure 2:**
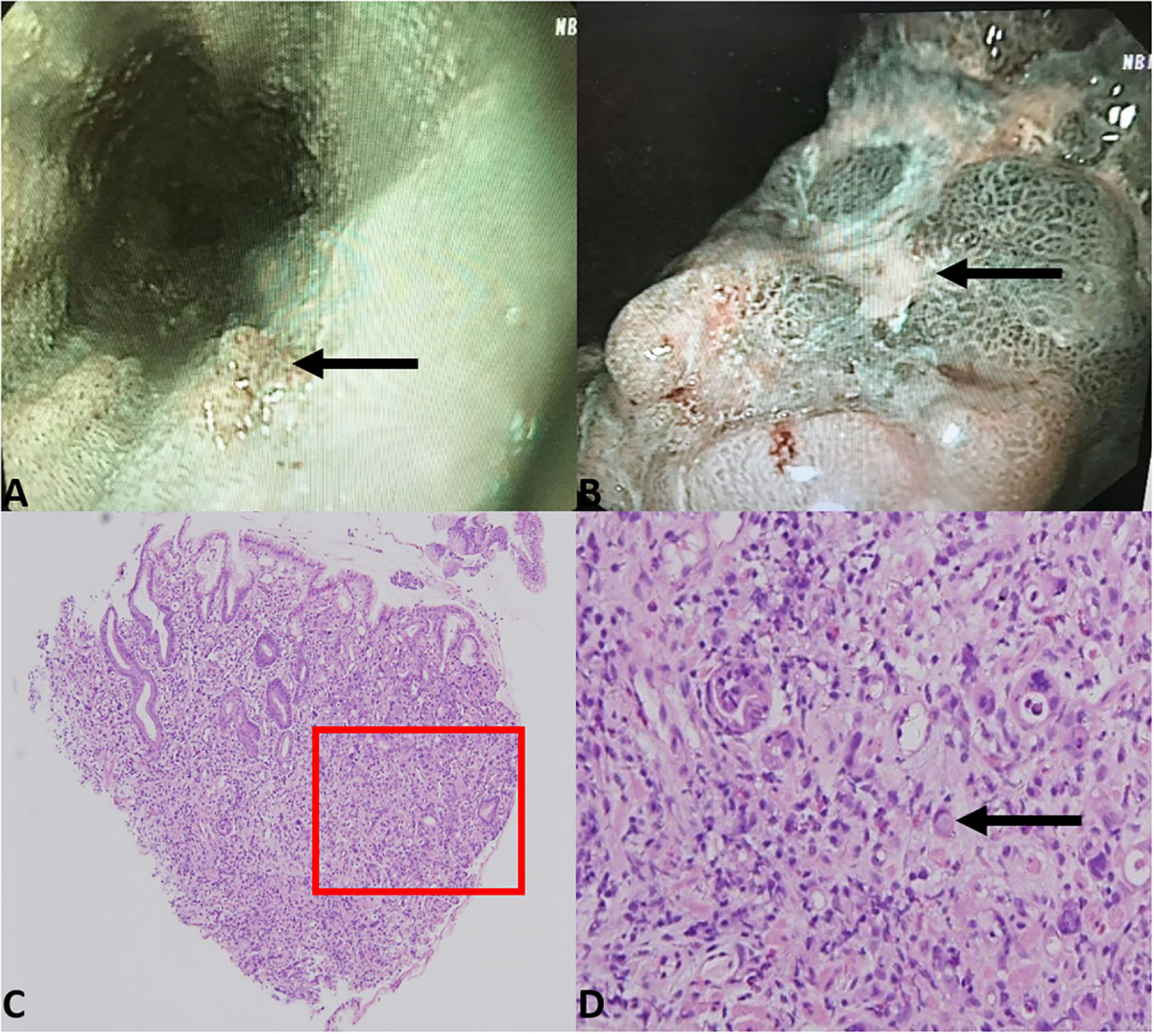
Gastroscope examination results showed papillary hyperplasia of the lower part of the esophagus **(A)** and rugged gastric angle posterior wall with ulcer **(B)**. Biopsy of the gastric angle revealed atypical tumor cells infiltrating the lamina propria of the gastric mucosa, with Lauren classification indicating a mixed type **(C)** 40× magnification). Localized signet ring cell carcinoma was observed **(D)** 200× magnification).

The patient underwent a systemic examination for underlying malignancy. Enhanced computed tomography results showed gastric wall thickening in the angle and enlarged lymph nodes in the lesser curvature, suggesting a possibility of gastric cancer (**Supplementary Figure S2**). Gastroscope examination results showed ulcers and papillary hyperplasia on the gastric wall and esophagus (**Figures 2A, B**). Histologic analysis of endoscopic biopsy confirmed gastric carcinoma with duodenal infiltration (**Figures 2C, D**). Immunohistochemistry results showed cytokeratin-7 (CK7) (+++), cytokeratin-20 (CK20) (+++), mucin-5 (MUC5) (+++), and caudal type homeobox 2 (CDX2) (+++), indicating primary gastric adenocarcinoma (poorly differentiated). Levels of serum tumor markers, including carcinoembryonic antigen (11.25 ng/mL, normal range: <5.0 ng/mL), carbohydrate antigen 19-9 (>1,000 U/mL, normal range: <37.0 U/mL), carbohydrate antigen 125 (165.30 U/mL, normal range: <35.0 U/mL), carbohydrate antigen 72-4 (57.87 U/mL, normal range: <6.9 U/mL), and tissue polypeptide antigen (2236.00 U/L, normal range: <120 U/L), were significantly elevated.

**Figure 3:**
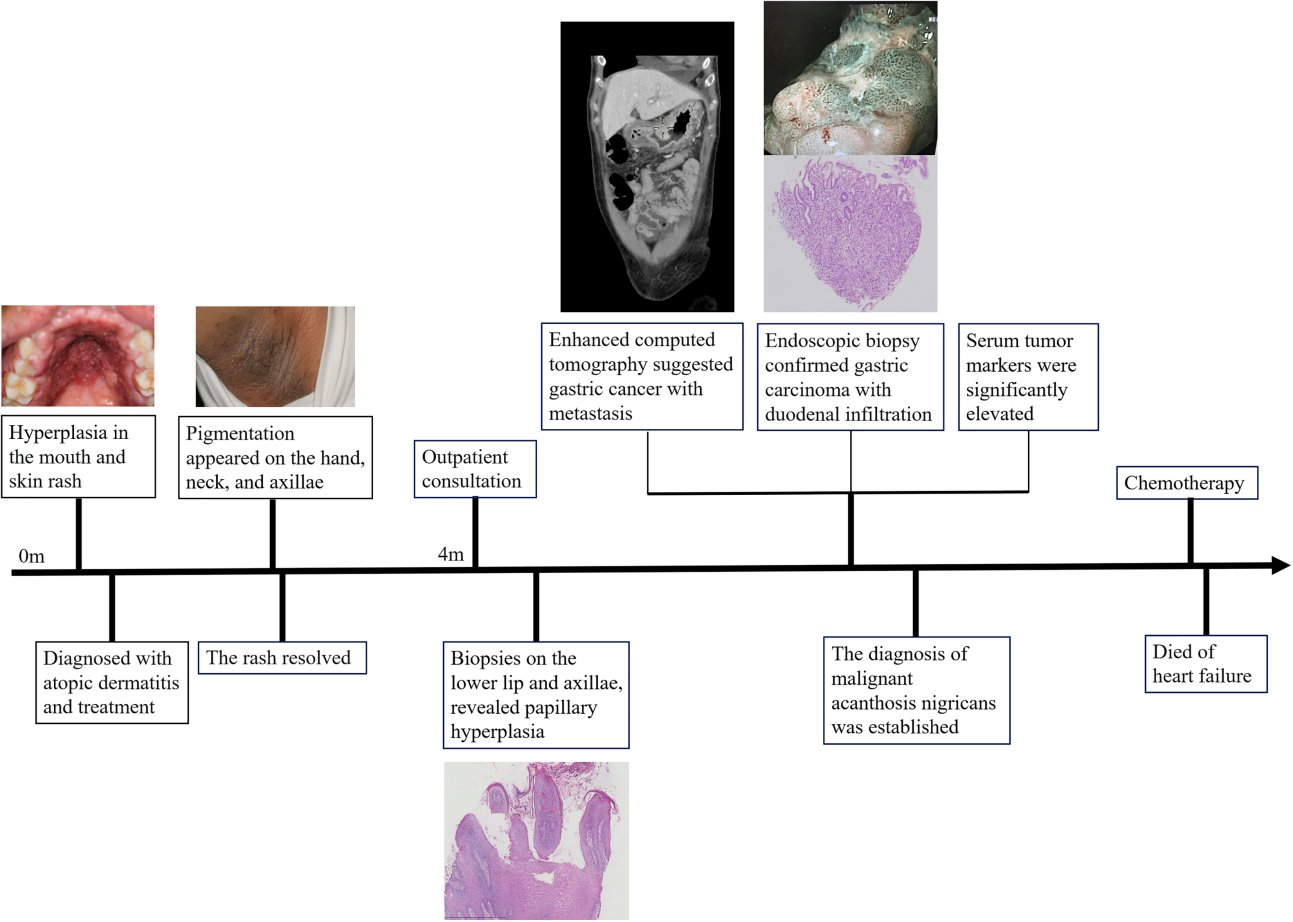
Timeline.

The diagnosis of malignant acanthosis nigricans was established. The patient received chemotherapy for the metastatic gastric carcinoma. Unfortunately, the patient died of heart failure during the treatment period. The timeline of the patient’s condition, medical visits, and outcome is presented in **Figure 3**.

## 3 Discussion

Malignant acanthosis nigricans in young populations is rare. In this report, we present a case of malignant acanthosis nigricans with multiple papillomatous and velvety oral lesions in a young female. Upon systemic examination, gastric cancer was detected. To the best of our knowledge, this is the first reported case of a young individual with malignant acanthosis nigricans presenting primarily with oral manifestations.

The differential diagnosis of acanthosis nigricans on oral mucosa holds particular importance for patient prognosis. Similar oral changes may occur in inflammatory papillary hyperplasia and HPV infection. Inflammatory papillary hyperplasia primarily presents as multiple small fibrous papillary growths, usually caused by wearing dentures and candidiasis, visible mainly on the denture-bearing hard palate (8). HPV-13 and HPV-32 infection can lead to multifocal, verrucous papules of the lips, buccal mucosa, and tongue (9), whereas HPV-6 and HPV-11 infections manifest as nodular or stalky masses with papillary surfaces (10). HPV tests help exclude these diseases.

Some skin disorders and syndromes also present with oral papillary lesions. Cowden syndrome (11) and ectrodactyly–ectodermal dysplasia–clefting syndrome (12) showed no evidence of skin hyperpigmentation. Linear epidermal nevus (13) and sebaceous nevus syndrome (14) can present both oral and cutaneous lesions resembling our case. The main point of differential diagnosis is that verrucous papules or pigmentation in these diseases appear in a linear configuration along skin tension lines. Focal dermal hypoplasia (15) is characterized by developmental disorders in multiple tissues and organs. Compared to this case, the above syndrome exhibits relatively small-range oral lesions, limited areas of skin lesions, and lighter pigmentation, with a tendency for family history. Specific genetic tests help make a correct diagnosis.

Extensive areas of papillary growth in oral mucosa and skin pigmentation in folds suggested that the patient might have acanthosis nigricans. Given that the benign and malignant forms of acanthosis nigricans can hardly be distinguished by mucocutaneous examination alone (2), we advised a thorough examination despite the young patient’s lack of discomfort, pain, or weight loss. This led to the discovery of gastric adenocarcinoma.

It is worth mentioning that Krawczyk (16) previously reported a case of papular rash with excessive keratosis, skin discoloration, and pruritus occurring simultaneously. Our patient also presented with a rash and was diagnosed with “allergy”. The presence of rash with excessive keratosis, oral papillary growth, and other manifestations should be carefully evaluated by clinicians to avoid overlooking the symptoms of malignant acanthosis nigricans.

Ramirez-Amador documented 12 cases of malignant acanthosis nigricans with oral manifestations prior to 1999 (17). Our literature review identified 23 additional cases since then. Nearly all reported cases involved patients older than 40 years. Papillomatosis emerged as the most prevalent oral manifestation, observed in 72.2% of cases, primarily affecting the lips. Among these malignancies, 42% were gastric cancers. Notably, 59.1% of patients exhibited oral lesions preceding the diagnosis of the malignancy. We present the cases in **Table 1** and summarize them in **Supplementary Figure S3**.

**Table 1:** Malignant acanthosis nigricans with oral manifestation literature search (1999–2024).

Reference	Age, years	Sex	Duration, months	Chief complaint	Oral mucosa		Cutaneous		Oral manifestation before/after cutaneous	Underlying malignancy	Diagnosis before/after malignancy detected
					Manifestation	Site	Manifestation	Site			
	30	F	4	Allergy	Papillomatosis and velvety hyperplasia	Lips, buccal mucosa, gingiva, hard palate mucosa, and tongue	Hyperpigmentation	Neck, axillae, and hands	Before	Gastric adenocarcinoma	Before
Liu et al., 2024 (18)	67	F			Velvety and papillary lesions	Upper lip, buccal mucosa, gingiva, and hard palate	Pigmentation	Around the mouth, neck, hands, and around the armpits		Ovarian cancer	Before
Al-Zahawi et al., 2024 (19)	58	F			Thickening	Lips and oral mucosa	Hyperpigmented and velvety thickening	Nose, neck, axilla, groins, palms, and soles		Gastric adenocarcinoma	After
Fayne et al., 2023 (20)	87	F	4	Verrucous papules	Verrucous papules coalescing into plaques	Upper lip, buccal mucosa, hard palate, tongue, and posterior aspect of the oropharynx	Verrucous papules coalescing into plaques	Left eyelid margin	After	Urothelial carcinoma	After
Yun et al., 2022 (21)	41	M	4		Hyperpigmentation	Lips	Hyperpigmented and pruritic plaques	Neck, axillae, and hands	After	Pancreatic neuroendocrine tumor	After
Zhang et al., 2021 (7)	71	F	4	Burning sensation	Hypertrophic lesions and papillomatosis	Buccal mucosa, gingiva, and tongue	Hyperpigmentation, thickened skin, and mild pruritus	Face, hands, feet, fold areas, and axillary and inguinal regions	After	Urinary bladder cancer	After
Rizwan et al., 2019 (22)	62	M	8		Velvety verrucous plaques	Lips, buccal mucosa, and hard palate	Hyperpigmentation, papular lesions, and velvety and hyperkeratotic plaques	Scalp, face, neck, lower abdomen, limbs, palms, and instep of soles		Hepatic ductal undifferentiated malignant neoplasm	After
Yu et al., 2017 (6)	74	M	7		Florid papillomatosis	Buccal mucosa	Hyperkeratotic plaques	Face, dorsal skin of fingers, and heels	Before	Gastric cardia tubular adenocarcinoma	Before
Wang et al., 2017 (23)	41	M			Verrucous lesions	Lips, buccal mucosa, and tongue	Hyperpigmented and thickened areas	Axillae and hands		Gastric adenocarcinoma	After
Karakas et al., 2016 (24)	55	M	3	Oral mucosal ulcers	Multiple mucosal oral ulcers		Hyperpigmented and velvety skin lesions	Lower and upper extremities, face, palms, axillary, inguinal, and anal regions		Squamous cell lung cancer	Before
Lee et al., 2015 (25)	70	F		Warty growths, oral discomfort, and difficulty eating	Papillomatosis	Lips, palate, and tongue	Verrucous or velvety pigmented plaques	Axillae, posterior neck, periumbilical region, and groin		Renal urothelial carcinoma	
Chu et al., 2014 (5)	59	F	3	Tumefaction lips	Scab and tumefaction	Lips	Hyperkeratotic and hyperpigmented skin, and velvety patchy lesions	Face, elbows, pudendum, groins, and axilla		Abdomen and pelvis metastases carcinoma	Before
Abu-Safieh et al., 2011 (26)	29	F	1		Blood oozing	Lips	Thickened, hyperpigmented, and warty skin	Axillae		Gastric adenocarcinoma	Before
Mignogna et al., 2009 (27)	74	F			Diffuse micropapillary lesions, “cerebriform” aspect	Inner upper lips, right cheek, and hard palate	Velvety rugose appearance	Palms and palmar surface of the fingers		Diffuse large B-cell lymphoma	Before
Krawczyk et al., 2009 (16)	44		2		Excessive keratosis and discoloration	Tongue	Excessive keratosis, skin discoloration, and pruritus	Trunk and axilla area	After	Gastrointestinal adenocarcinoma	After
Canjuga et al., 2008 (28)	78	F			Papillomatous growth	Lips, buccal mucosa, and hard palate	Verrucous changes and tiny pigmented macules	Right ear auricle		Bladder papillary transitional cell carcinoma	After
Schnopp et al., 2007 (29)	44	F	15		Papillomatosis, thickened and furrowed changes	Lips, buccal mucosa, and tongue	Darkening skin, small and velvety plaques	Neck	After	Gastric adenocarcinoma	Before
McGinness et al., 2006 (30)	81	F		Burning and soreness	Verrucous papillomatous papules and plaques	Lips, buccal mucosa, and tongue	Accentuation	Palms	Before	Pancreatic adenocarcinoma	Before
Pentenero et al., 2004 (31)	53	M		Sore palate	Thickened mucosa with a velvety and papillomatous surface	Lips, buccal mucosa and palate	Velvety rugose appearance, hyperkeratotic verrucous, pigmented, and brownish papules	Palms, back, and axillae		Gastric adenocarcinoma	After
Cairo et al., 2001 (32)	73	M		Burning sensation and pain	Papillomatosis	Vestibular mucosa, gingiva, palate, and dorsum of the tongue	Hyperpigmented and thickened skin	Neck, axillae, and the internal surface of the thighs		Lung carcinoma	Before
Scully et al., 2001 (33)	69	F	9	Painless gingival lesions	Swelling, increased stippling, and diffuse thickening	Lower labial mucosa, gingival, and palate but not the edentulous areas	Slightly keratotic, warty lesions, and florid seborrhoeic warts	Palms, soles, axillae, and proximal limbs		Cholangiocarcinoma	
Yeh et al., 2000 (34)	69	M	6		Papillomatous involvement		Thickened and hyperpigmented skin, papillomatous involvement, and seborrheic keratoses	Face, nape of the neck, axillae, groins, limbs, and trunk		Gastric adenocarcinoma	Before
Bottoni et al., 2000 (35)	80	M	12		Papillomatous and verrucous lesions	Lips and oral mucosa	Diffuse brownish hyperpigmentation, velvety thickening, and warty lesions	Neck, axillae groins, and limbs		Non-small cell lung carcinoma	Before
Ramirez et al., 1999 (17)	55	F	12	Sore palate	Painless extensive papillomatosis	Upper vermilion and lip mucosa, buccal mucosa, gingiva, palate, and dorsum of the tongue	Hair loss	Axillary, palpebral, and scalp		Gallbladder adenocarcinoma	Before

In conclusion, a significant percentage of malignant acanthosis nigricans patients exhibited oral lesions prior to the detection of the underlying malignancy. Therefore, clinicians, especially oral healthcare professionals, should remain vigilant. Detailed differential diagnosis is necessary, and a comprehensive systemic examination is required to facilitate early diagnosis of malignancies and improve patient prognosis. Furthermore, in contrast to previous cases, our patient was only 30 years old and considered herself to be in a healthy condition. This suggests that age may not be a restrictive factor for malignant acanthosis nigricans. On the other hand, the rash may coexist with skin and mucosal lesions, warranting that differentiation from allergies and clinical observations of mucosal manifestations after the regression of the rash are important.

## Data Availability

The original contributions presented in the study are included in the article/**Supplementary Material**. Further inquiries can be directed to the corresponding author.
