# Supplementary material for: Malignant acanthosis nigricans with oral manifestations in a young female: a case report and literature review
Source: Front Oncol. 2024 Sep 24;14:1459148. doi: 10.3389/fonc.2024.1459148 (PMC11458928; doi:10.3389/fonc.2024.1459148)
Supplement: Supplementary file 1 [file DataSheet1.docx]

Supplementary Material

#
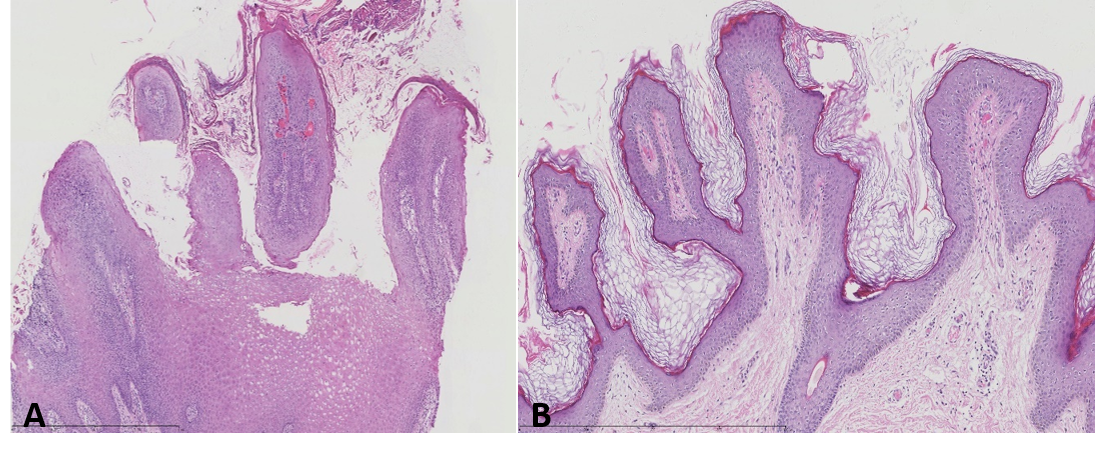
Supplementary Figures

**Supplementary Figure S1.** Histological picture of lesions on lower lip **(A)** and skin of axillae **(B)** showing papillary hyperplasia.


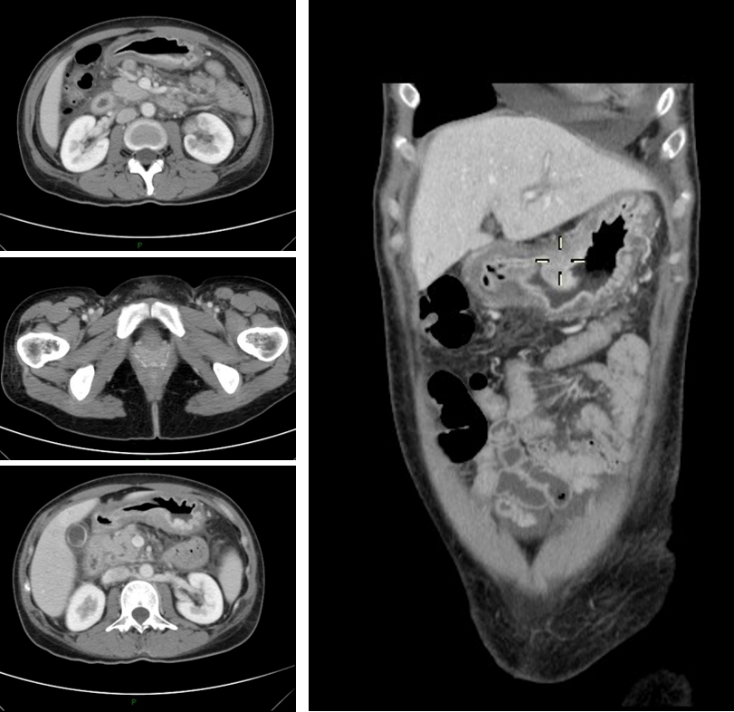


**Supplementary Figure S2.** Enhanced computed tomography showed gastric wall thickening in the angle, enlarged lymph nodes in the lesser curvature, possible gastric cancer; a mass in the lower pole of the left kidney, possible metastasis; bilateral adrenal gland enhanced nodules, possible metastasis; multiple mesenteric lymph nodes of undetermined nature; abdominal pelvic effusion; bilateral pleural effusion with atelectasis of left lower lobe; pericardial effusion.


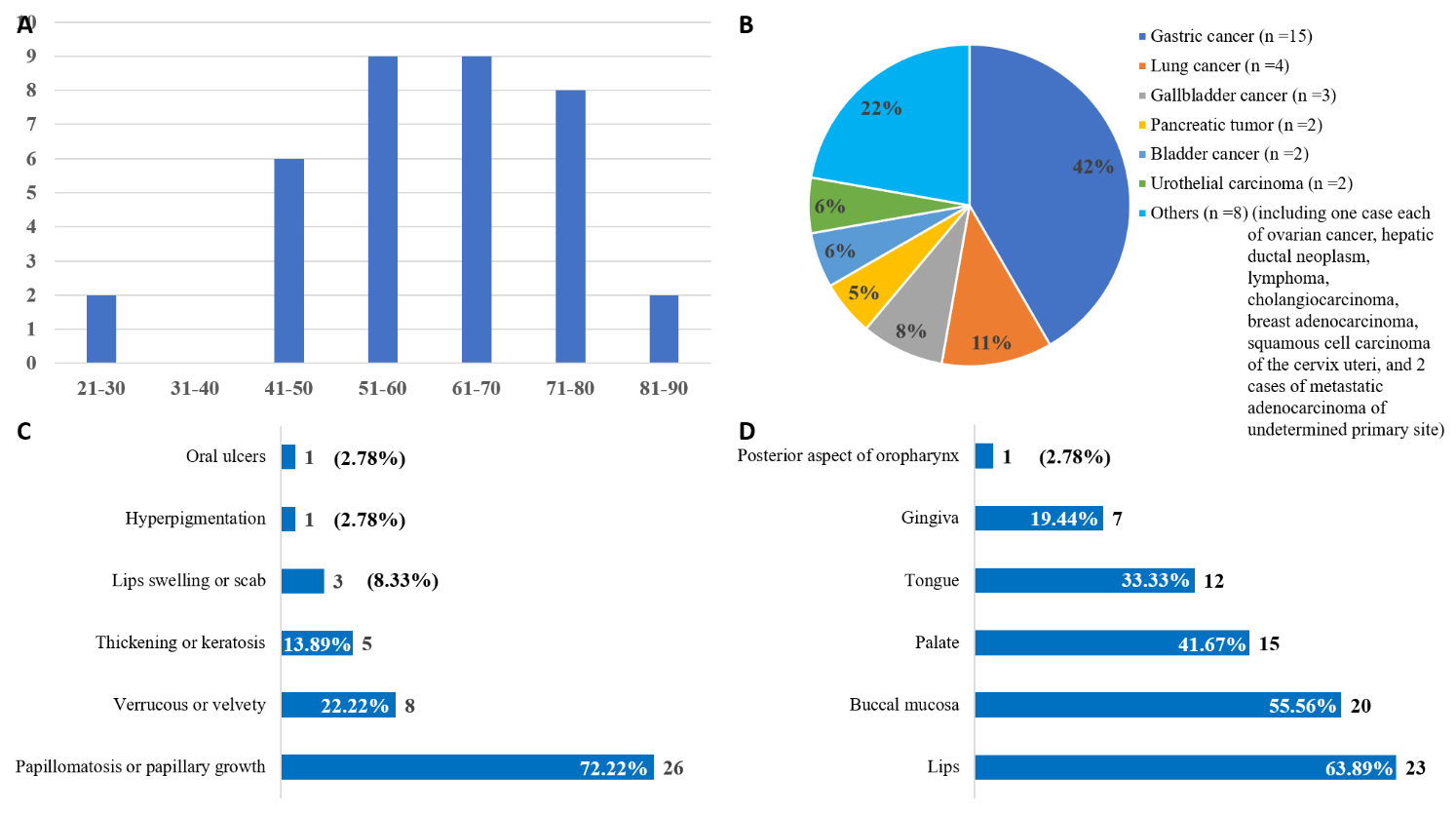


**Supplementary Figure S3.** Incidence of malignant acanthosis nigricans in different age groups **(A).** Associated underlying conditions **(B)**. Incidence of different types of oral mucosal lesions **(C)**, and involved oral anatomical sites **(D)** in malignant acanthosis nigricans.
